# Preclinical models as patients’ avatars for precision medicine in colorectal cancer: past and future challenges

**DOI:** 10.1186/s13046-021-01981-z

**Published:** 2021-06-05

**Authors:** Erika Durinikova, Kristi Buzo, Sabrina Arena

**Affiliations:** 1 Candiolo Cancer Institute, FPO – IRCCS, Strada Provinciale 142, Km 3.95, 10060 Candiolo, TO Italy; 2 Department of Oncology, University of Torino, Strada Provinciale 142, Km 3.95, 10060 Candiolo, TO Italy

**Keywords:** Colorectal cancer, Personalized medicine, Preclinical models, Xenolines, Patient-derived xenografts, Zebrafish patient-derived xenografts, Organoids

## Abstract

Colorectal cancer (CRC) is a complex and heterogeneous disease, characterized by dismal prognosis and low survival rate in the advanced (metastatic) stage. During the last decade, the establishment of novel preclinical models, leading to the generation of translational discovery and validation platforms, has opened up a new scenario for the clinical practice of CRC patients. To bridge the results developed at the bench with the medical decision process, the ideal model should be easily scalable, reliable to predict treatment responses, and flexibly adapted for various applications in the research. As such, the improved benefit of novel therapies being tested initially on valuable and reproducible preclinical models would lie in personalized treatment recommendations based on the biology and genomics of the patient’s tumor with the overall aim to avoid overtreatment and unnecessary toxicity. In this review, we summarize different in vitro and in vivo models, which proved efficacy in detection of novel CRC culprits and shed light into the biology and therapy of this complex disease. Even though cell lines and patient-derived xenografts remain the mainstay of colorectal cancer research, the field has been confidently shifting to the use of organoids as the most relevant preclinical model. Prioritization of organoids is supported by increasing body of evidence that these represent excellent tools worth further therapeutic explorations. In addition, novel preclinical models such as zebrafish avatars are emerging as useful tools for pharmacological interrogation. Finally, all available models represent complementary tools that can be utilized for precision medicine applications.

## Background

Colorectal cancer (CRC) represents the third leading cause for cancer-related death in the western world [1]. While 5-year survival rates are estimated to be between 85 and 90% for patients with localized colorectal cancer, they dramatically decrease to ~ 12% in patients with advanced-stage disease [2]. Thus, the metastatic disease is an important clinical challenge that requires a need to discover new therapeutic strategies driven by solid preclinical evidence. The standard of care for metastatic CRC consists of medical treatment using chemotherapy and targeted therapy with the final aim of maximizing shrinkage of the tumor and suppression of further tumor growth and spread, accompanied by locoregional treatment whenever possible [3–6]. Even though decades of efforts devoted to increase cancer patients’ survival brought major advances, this area is still in medical need for new therapeutic options. One of the major obstacles to developing novel and efficient regimens for treatment of patients is the challenge to translate scientific knowledge from bench to bedside. Many drugs are initially successful in cancer laboratory models but fail in clinical trials [7, 8], and many clinical trials failed due to inappropriate patient selection [9, 10].

The establishment of preclinical models, which faithfully recapitulate CRC pathogenesis, represents a key tool for testing novel treatment options that could provide long-term benefits for the treatment of CRC patients. Considering both the huge effort spent on preclinical studies and the costs of clinical trials, novel therapeutic strategies should be carefully designed to offer the highest model predictive accuracy coupled with the saving of time and resources.

**Fig. 1:**
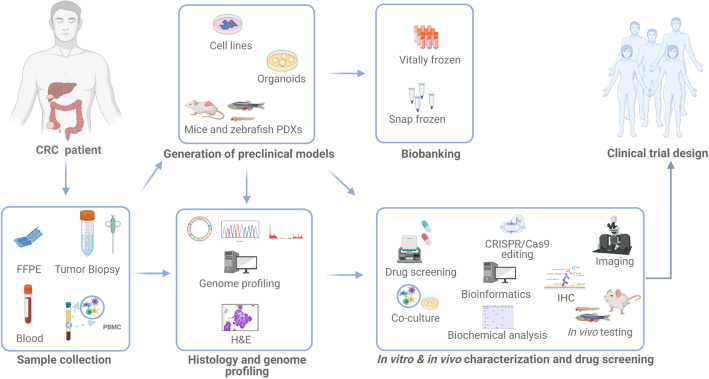
Generation and applications of CRC preclinical models. After CRC patient tumor’s surgery or biopsy, the blood sample (PBMC is obtained after sample processing) and the tumor specimen are collected. These biological materials are subsequently used for histopathological analysis along with genetic profiling as for medical therapeutic decision in case of metastatic colorectal cancer. In parallel, preclinical models such as primary cell lines, XLs, PDXs, zPDXs and PDOs can be generated in the laboratory. Once established, these models are expanded in order to create sufficient material for storage and biobanking. Multiple applications can be performed for in vitro and in vivo characterization of these models. The integration of these results, together with bioinformatic analysis, can be finally potentially translated to the design of novel clinical trials. PBMC, peripheral blood mononuclear cell; XLs, patient-derived xenolines; PDXs, patient-derived xenografts; PDOs, patient-derived organoids; FFPE, formalin-fixed paraffin-embedded; H&E, hematoxylin and eosin; IHC, immunohistochemistry. This figure was created with BioRender.com

To best provide benefits to future healthcare of oncologic patients, the individual treatments for personalized medicine are in demand. We are in the era of unprecedented opportunity to use different types of preclinically available models and techniques to conduct laboratory studies with the overall aim to model disease “at the bench”, to unravel significant genetic, transcriptomic and proteomic players taking part in initiation and progression of the tumor, and to include the identification of anticancer agents with improved translational potential leading to precision medicine. An ideal preclinical CRC model should not only show close histological similarity to the tumor of origin and maintain druggable genomic alterations for targeted approaches, but should also address practical issues, such as easy handling and good in vitro and in vivo growth characteristics. The discovery and testing of novel strategies have been conducted using in vitro*, ex-* and in vivo models. Human cancer-derived cell lines have historically provided important contributions to the understanding of the biology of cancer [11] and of essential mechanisms within tumor cells. In recent years, a plethora of preclinical models has been developed. Primary cell cultures derived from patient biopsies, patient-derived xenograft models (PDXs), both in mice and in zebrafish (zPDXs), PDX-derived cell lines (xenolines, XLs), or three-dimensional organoid cultures became a breakthrough for expansion of vital tissue, and are decisive for applied research and therapeutic studies [12–14]. Virtually, these patient-derived tumor models constitute a valuable, amplified source of material for both analysis of biological characteristics as well as for predicting drug response (Fig. 1), although they can also present few limitations. In this review, we will discuss the strengths and the challenges of available experimental preclinical models of CRC, but also caveats and drawbacks attributed to each model. We will highlight the most important studies and illustrate how they can be used to address missing gaps within CRC cancer research. Finally, we will focus on translational purpose of individual models and discuss their potentials and new directions eventually leading to personalized medicine as the ultimate goal in molecular oncology.

### Patient-derived xenografts (PDXs)

**Fig. 2:**
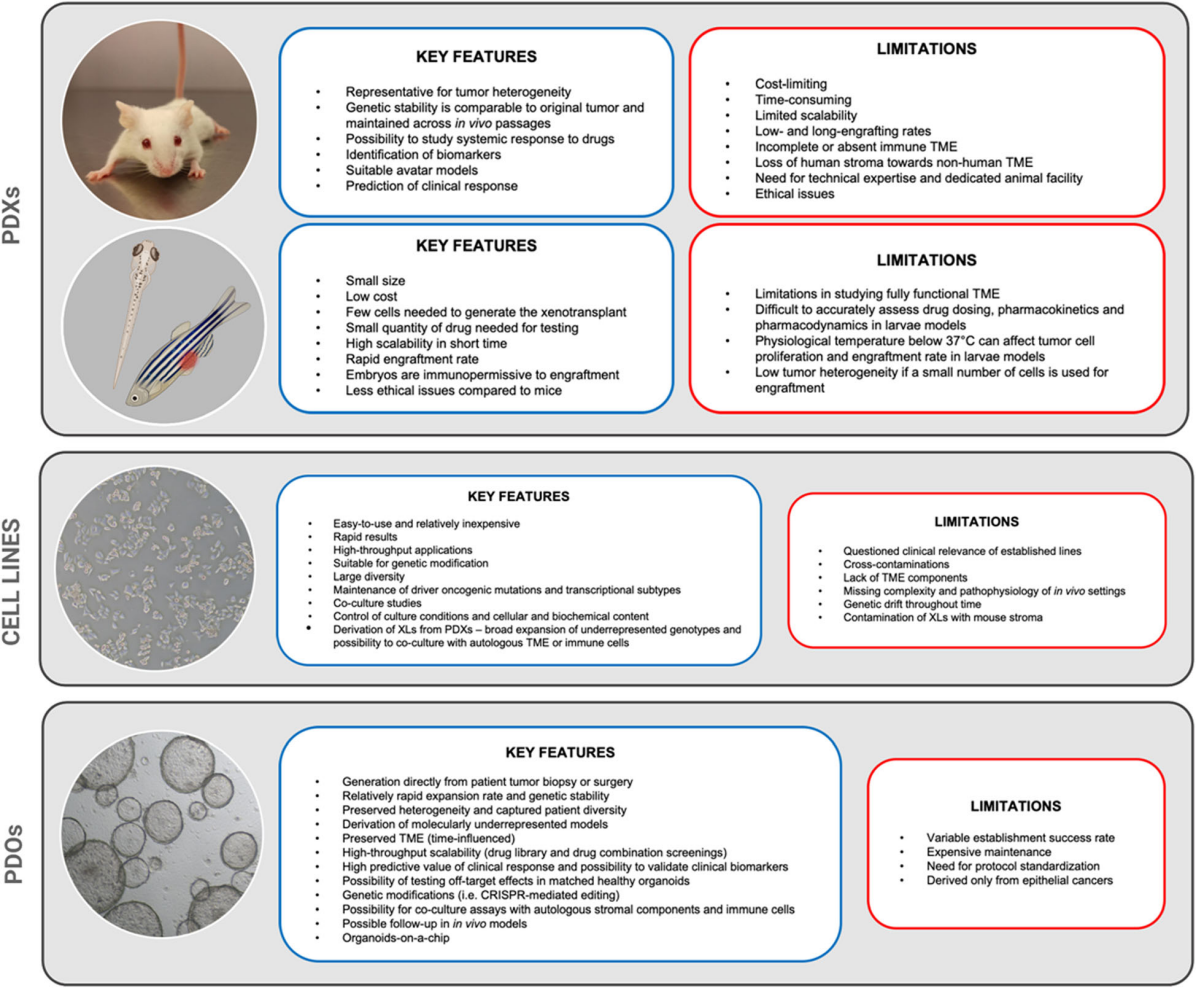
Summary list for the main characteristics of individual CRC preclinical models. Features are divided into model-favouring key features and limiting factors. PDXs, patient-derived xenografts; PDOs, patient-derived organoids; XLs, xenolines; TME, tumor microenvironment. This figure was created with BioRender.com

Tumor cell transplantation has become a colossal experimental tool to assess the malignant phenotype. After surgical resection or biopsy of the primary tumor or metastasis, CRC patient specimens undergo clinical analyses such as histology and immunohistochemistry along with molecular testing when requested. Provided the patient’s consent, each tumor sample can be subjected to oncologic research and directly processed for genomic analyses or long-term storage by cryopreservation [15]. Further in vitro manipulation can comprise the establishment of either primary cell culture or organoids, or the subcutaneous transfer as a fresh specimen into immunocompromised mice for the growth and establishment of patient-derived xenografts (PDXs). Throughout the past decades, different types of murine PDX models have been developed to study CRC tumorigenesis, metastatic spread, immune response, and testing of novel drug combinations [16, 17]. On the other hand, there are also different non-mammalian-based models established for cancer research. Naturally immunodeficient chick embryo chorioallantoic membrane (CAM) models serve mainly for angiogenic activity and invasion assays with evaluation of metastatic potential of tumor cell lines or primary tumor tissues [18–20]. An alternative in vivo PDX model proving to be effective and being comparable with mouse xenografts and human organoids is zebrafish [14]. Zebrafish PDXs (zPDXs) can be created by transplantation of cancer cells from either solid tumors or blood malignancies in 48-h post-fertilization larvae or in adult genetically immunocompromised zebrafish since early 2000s [21–26]. zPDXs have been applied in a variety of oncology research, mainly in the establishment of diverse tumor models by xenotransplantation or carcinogenic chemical and genetic technology, in the assessment of tumor angiogenesis and metastatization, and in drug toxicity evaluation along with screening assays, as it is in more details reviewed in [14, 27, 28]. Due to very low cost of the husbandry and maintenance, together with vast breeding capability, large chemical screens have been performed in zebrafish, such as anti-melanoma chemical genetic screen [29], anti-leukemia compound screen [30], anti-angiogenic [31] and anti-lymphatic drug screens [32]. zPDXs models have been constantly undergoing “technical” developments such as generation of immunodeficient zPDXs grown at physiological temperatures which are optically-clear and enable dynamic visualization of tumor cells at single cell resolution with tumor growth kinetics and histopathology alike to those grown in immunodeficient mice [24]. The first proof-of-concept study with CRC in zebrafish larvae with developed protocol of utilizing resected tumor specimens from patients for larvae engraftment was demonstrated by the group of Dr. Godinho Ferreira [33]. In spite of the small cohort of 5 patients only, they showed 100% donor engraftment success. Subsequent treatment of zPDXs with standard-of-care combination revealed concordant responses between in vivo models and actual patient clinical response in 4/5 cases to FOLFOX, and in 3/5 cases to cetuximab. As in the case of previous study, 4 days were sufficient to distinguish responder and non-responder to radiotherapy of zPDXs with rectum cancer samples. Even though the patients’ number was limited, this proof-of-concept experiments together with large preclinical testing demonstrated feasibility of using zebrafish larvae as avatars to potentially predict clinical response to neoadjuvant therapy in only 12 days of experimental flow [34]. This would represent a major clinical advancement to set up real-time co-clinical trials, but as critically emphasized, the applicability leading to clinical decision-making need to be addressed in larger cohorts. Zebrafish features such as ease of genetic manipulation of embryos, logistic advantages of scale, cost, time and high-throughput applications with the potential for automation, less ethics constrains, and need of small number of transplanted cells or reduces amounts of drugs per test render them advantageous for precision therapy (Fig. 2). Still, issues like human physiological temperature adaptation, short window of immune incompetency during larval development, type of drug treatment and potential toxic effects of anticancer drugs on larvae itself, plus lack of patient-specific immune tumor microenvironment (TME) can be crucial for their full translation to the clinic, although recent seminal studies are posing promising bases to understand mechanisms related to innate immune response in CRC [35].

Conservatively, the most successful and translationally relevant procedure has been the expansion of human vital tissue by xenotransplantation in immune-deficient mice. Engraftment of the tumor fragment preserves the vital cell-cell interactions, with the caveat that the dynamics of the human TME has been replaced by mouse components. Although PDXs generally retain the histopathological features of the tumor from which they are derived, overall tumor heterogeneity might be affected since they are obtained from spatially different pieces of the same tumor and may consequently hold different genetic features affecting the course of the disease in the laboratory [36, 37]. To preserve heterogeneity of the patient sample, either multiple pieces from different tumor regions could be implanted or injection of a cell suspension from the whole processed tissue might be performed [38, 39]. PDXs recently proved to be a reliable model for in-depth investigation on tumor heterogeneity in its spatial context using metastatic CRC [40]. Several extensive studies indicated that PDXs recapitulate molecularly defined subtypes within the CRC. Patients can be stratified into different gene expression-based consensus molecular subtypes (CMS) assigned with specific biological features defined by their heterogeneity and possible clinical outcomes [41, 42] and then extrapolated into potential treatment options with subtype-specific drugs [43]. The study by the group of Dr. Medema [44] identified that inter-patient heterogeneity within the context of CMS is not equally represented in CRC PDX models, pointing to the issue of CMS2 major subtype being strongly underrepresented due to lower engraftment success. In general, one of the main challenges in working with PDXs is the limited engraftment rates that can depend on different clinical parameters, such as stage of the disease, tumor localization, molecular subtype, microsatellite (MSI) status, and genetic alterations [12, 45]. More recently, researchers have attempted to better discriminate the stromal contribution to the CRC transcriptome by using a novel cancer cell-gene expression classifier, which has been developed and applied to a vast collection of PDXs. This analysis led to the identification of distinct transcriptional CRC intrinsic subtypes (CRIS) which distinguishes between the effects of stromal infiltration. Importantly, this transcriptional classifier might have further prognostic and predictive potential for patients’ management [46].

PDXs can also diverge from the original tumor leading to clonal selection [39, 47, 48], thus raising the question of whether only early-passage PDXs should be used for translational biology. Considering that more aggressive tumors have a higher success rate in PDXs establishment, it is possible that the fitter clones can expand and become more dominant, and may represent the ones which would be selected under the treatment pressure in patients, thus contributing to progression of the disease [47, 49]. To fully elucidate genomic evolution or stability of PDXs during engraftment and propagation, comprehensive analyses are needed. Initial studies have raised the concern that PDX passaging might lead to selection of pre-existing subclones and accumulation of copy number alterations (CNA) potentially altering the response to treatment [48]. More recent results coming from an in-depth international study encompassing the largest collection of CNA datasets of PDXs of multiple passages demonstrated conservation and high degree of molecular fidelity of PDXs to the original tumor, as well as PDXs across increasing passages [50]. This extensive effort pointed at the increasing trend in academia and industry to establish an international and interdisciplinary network for gathering and sharing PDX models, namely EurOPDX and PDXNET consortium [49–52]. It is imperative on a global basis to standardize the methods for sample processing and data collection with the aim to collaborate on preclinical and co-clinical trials [53]. As cancers are highly heterogeneous at the genomic level, data resources shared in these networks can provide necessary information by comparing clinical samples with samples in the databases, and likely identify personalized treatment plans.

Over the last decade, various studies demonstrated that established xenografts are useful for evaluation of new drugs and their combinations, and drug responses of the PDXs were directly correlated to the response of the patients from whom the tumors were obtained [54]. This high concordance strongly supported the use of clinically relevant PDX models for investigation of therapeutic targets also within CRC. Considering that *RAS* mutational status is the only FDA-approved biomarker for anti-EGFR treatment in CRC, most studies focused on uncovering of this pathway. The initial work focused on identification of novel biomarkers of resistance to the anti-EGFR monoclonal antibody cetuximab was reported in a cohort of 85 metastatic CRC PDXs [55]. The study was able to stratify PDXs into responders and non-responders with rates corresponding to those observed in the clinic, thus anticipating clinical findings. This first proof-of-concept study detected HER2 amplification as a new biomarker of resistance to EGFR inhibition as well as a positive predictor of response to anti-HER2 therapy. Long-lasting tumor regression was observed after combined inhibition of EGFR and HER2 in cetuximab-resistant, *KRAS-, NRAS-, BRAF-, PIK3CA*-wt, and *HER2*-amplified metastatic CRC [55]. Henceforth, the further expansion of the knowledge between the connection of HER2 and CRC led to the identification of HER2-activating mutations also causing resistance to EGFR monoclonal antibodies and provided new intervention possibilities using dual anti-HER2 targeted therapy for the management of patients suffering from *HER2*-mutated *KRAS*-wt CRC [56, 57]. These studies suggested inclusion of *HER2* gene sequencing in metastatic CRC routine testing. Not long after, the first trial evaluating the efficacy of combination of trastuzumab and lapatinib as dual anti-HER2 strategy in HER2-positive CRC heavily pretreated patients proved its benefits [58–61]. Taken together, as *HER2* amplification became a clinically relevant genetic alteration occurring in 3–5% metastatic CRC patients [58], it is imperative to understand the molecular determinants of dual HER2 blockade resistance which may strike as primary or acquired resistance [37, 62–64]. This understanding might in time lead to identification of further combinatorial treatment options with different set of targeting agents. One example comes from the data of lung cancer where co-treatment with irreversible pan-HER inhibitors or the newest antibody-drug conjugate trastuzumab deruxtecan T-DXd demonstrated potent effects. This could be potentially translated to other types of HER2-driven cancers in near future [65]. Based on these results and many others, we can conclude that CRC PDXs play an important role in identifying biomarkers that could lead to better stratification of patients with assessment of drug efficacy in this pre-selected population, and thus could facilitate optimization of clinical trials’ design, especially of those that are at early phase.

Identification of new druggable targets and establishment of novel animal models within CRC is still actively ongoing. Orthotopic PDX model of metastatic tumors in liver parenchyma with subsequent successful propagation has been established, thus opening the clinically relevant possibility to further extend the research not only to models of primary tumor, but also of metastatic disease [66]. Another example of a very recent model establishment is the first unique PDX model of rectal cancer derived from patients’ samples collected prior to the initiation of therapy, which supported the translational applicability of the PDX platform. This disease-specific model reproduced the response of corresponding patients, and demonstrated the enhancement of 5-fluorouracil/radiotherapy efficacy by cetuximab [39]. Moreover, PDX models provide not only experimental proof that some drugs should not be used in certain clinical settings [67], but they also serve to uncover novel promising therapeutic agents. One example is pevonedistat, a selective inhibitor of the ubiquitin-like NEDD8 conjugation pathway. This inhibitor showed promising results in clinically aggressive histotypes of poorly differentiated and high-grade mucinous CRC which also carried *KRAS*- and *BRAF*-activating mutations, representing an unmet medical need, and uncovered cadherin-17 as a valuable negative predictor of CRC sensitivity to this inhibitor [68]. Detailed listing of more studies is out of the scope of this review. Another large focus in the field of oncology is the immuno-oncology. Immunotherapy represents an effective strategy for tumor eradication and in a subset of metastatic CRC patients with mismatch-repair-deficient and microsatellite instability-high disease, these therapies achieve long-term durable remission, thus highlighting the immense promise of immunotherapy for CRC [69]. The detailed insight into this approach is beyond the scope of this review since immunocompetent mice are involved and this topic is more precisely discussed elsewhere [70–73]. However, the development of humanized mice carrying an autologous human tumor and immune systems will increase the clinical relevance with the overall aim of personalized medicine studies. In the meantime, the field of immunotherapy constantly proceeds further to novel experimental approaches. For example, testing of engineered HER2-specific CAR-T cells (T cells engineered with a chimeric antigen receptor) showed both regression and elimination of HER2-positive CRC tumors in a PDX model and subsequently protected mice from tumor re-challenge [74], as well as exploitation of combinatorial immunotherapies, several of which are currently under clinical evaluation [75].

All the studies discussed above support the use of personalized PDX models, so-called “avatars”, as a powerful investigational platform for actionable clinical decisions in “co-clinical” trials. This paralleled study between patients and PDX models in real time coupled with tumor genomic profiling might help to tailor treatment strategies based on individual cancer vulnerabilities, as well as to identify resistance mechanisms appeared during the clinical treatment, and to explore the susceptibility for novel drug combinations with the overall goal of overcoming resistance [76–78]. Eventually, avatars could create an evidence-based rationale for new clinical trials as this concept has the potential to revolutionize health care process. To the contrary, it is crucial to point out that this concept is endowed also with important drawbacks. For some tumor types the engraftment phase requires larger amount of tumor material to increase the take rate, and many implants still fail. It is also quite expensive and very time-consuming, and when thinking of a patient with advanced disease, waiting several months for PDX establishment is not an option. Even so, for the patients in whom the personalized treatment was well suited based on results obtained thanks to the avatar model, the clinical activity could be exceptional.

During propagation of PDXs in vivo, an important limitation for studies comprising TME or agents targeting immune system is the drift of human stromal components towards mouse ones [47, 79–82], and the already mentioned lack of immune system. In addition, it has been shown that serial transplantation of CRC can be affected by minor numbers of residual EBV-infected B-lymphocytes leading to B-lymhoproliferations, thus stressing the need of repeated phenotypic testing of serial PDXs [83]. All these pitfalls need to be carefully taken into consideration when designing our study, and make large-scale screenings limited. In vitro 2D or 3D culture models, as described further, could serve for high-throughput screenings, and PDX platform should be viewed mainly as complementary to these preclinical models with the potential of validation of selected targets in vivo.

### Two-dimensional (2D) cell line models

The traditional approach for understanding of tumorigenesis, cancer biology and drug discovery has historically involved human cancer-derived cell lines. This fundamental cancer model has been used to decipher molecular and phenotypic features of cells with the aim to test translational hypotheses and providing genome-drug response correlates. Established cell lines are relatively easy and inexpensive to use and provide rapid experimental results. The pioneering works linking drug sensitivity with genotype data are associated with NCI60 cell line panel [84, 85], rapidly recognized as a rich source of information and still representing one of the most commonly exploited resources for pan-cancer studies. Further efforts have allowed for the establishment of larger cell line collections to reveal context-specific dependencies in cancer cells as well as identify rare drug-sensitizing genotypes [86]. Datasets such us the Cancer Therapeutic Response Portal (CTRP) [87] and Cancer Cell Line Encyclopedia (CCLE) [88] have been instrumental for the identification of novel potential cancer vulnerabilities, and further development has recently allowed for a more comprehensive characterization [89]. Up to date approaches such as CRISPR-Cas9 screen [90, 91] and investigation of mutational signatures over extended periods of time [92] clarify the cancer biology more deeply than ever and contribute to new, diverse and likely effective portfolios of cancer drug targets. Looking specifically at CRC, one primary study was focused in the Bardelli’s laboratory on 151 established CRC lines [93] representing molecular heterogeneity in terms of oncogenic mutations and transcriptional subtypes previously defined in CRC patients. The authors optimized this cell platform to perform comparative drug response assays and identify novel CRC dependencies on kinases for which clinically approved drugs are available.

Further efforts from the same group have led to a more extended CRC cell line bank comprising established lines together with xenopatient-derived cell lines or xenolines (XLs), as discussed later, to perform genomic [62] and drug response [62, 94] analyses. A complementary study performing longitudinal analysis of cell lines and PDX models [95] highlighted that microsatellite instable (MSI) and *POLE*-mutated CRC models evolve more rapidly respect to microsatellite stable (MSS) models during time due to generation of novel SNVs and frameshifts potentially giving rise to the acquisition of neoantigens, that constitute an attractive target for immunotherapies. During evolution of cancer, tumor cells can indeed produce neoantigens, arising from acquired genetic alterations, that could trigger immune response [96, 97], although, this study pointed at the imperfection of in vitro models used due to no presence, and consequently no impact, of the immune system for the testing of evolutionary immune-dynamics of the tumor.

Established CRC cell lines have extensively undergone proteomic analysis, showing that they are representative of primary tumors even though systematic differences between cell lines and tumor proteomes were apparent and attributed to tumor stroma, extrinsic signaling and different growth conditions, thus underscoring both the pros and cons of cell line models unraveling biology of the tumor [98]. As such, even though established cancer cell lines are still the mainstay due to easy manipulation, global studies and high-throughput applications, the clinical relevance of this model has been continuously questioned as there was no correlation with the clinical samples that they are supposed to model [99, 100]. This brings us to several important drawbacks and limitations when thinking of using 2D cell lines. They represent a population of cells kept long-term in in vitro conditions, that were naturally selected to yield relatively homogeneous cell populations, and thus likely differ from the original tumor substantially as they may lose characteristics close to the patient [101]. These models also do not account for the complete complexity and pathophysiology of in vivo tumors and reports of cross-contaminations have been published as well. On the other hand, it is important to stress the fact that these limitations should not hide the extensive data generated by cell lines, fostering the development of more in vitro preclinical models and validation in clinically relevant models the results previously observed in cell lines.

As tremendous amounts of information have accumulated regarding the diversity of molecular changes in CRC, additional models closely resembling genomic alterations in primary tumors and metastasis are needed. Ultra-low passage CRC primary cultures are the basis for modern preclinical research. Several world laboratories succeeded in generating patient-derived CRC primary cultures not only from primary tumors, but also metastases, with their subsequent phenotypic, genomic and drug response profiles characterization [102–106]. CRC primary cultures were shown to express a large diversity of mutation spectra and gene expression profiles [107], hold prognostic information for predicting peritoneal metastasis seeding [108], were tested in combinatorial treatments [109] and used for preparation of chemo-resistant counterparts for studying cancer stem cells and resistance to conventional treatment [110]. Also, novel targets may be identified in cancers which are poorly represented such as early-onset CRC (eoCRC) [111]. Primary cultures consume less time than PDXs establishment and were proven to be more representative of the genetic diversity and heterogeneity when compared to established cell lines, reflecting the original molecular signature of the tumor they were established from. Though, as shown by the group of Dr. Linnebacher [12, 104] and others [112, 113], primary CRC cultures are difficult to maintain. The establishment success rate is approximately 10% and the clonogenic capacity is lost after several passages as cells undergo massive death.

In parallel to a challenging primary cell culture attempt, the patient tumor tissue can be engrafted into immunocompromised mice for the establishment of PDXs which can be further processed into derivation of so-called xenopatient-derived cell lines or xenolines (XLs). This procedure yields approximately double the success rate compared to CRC primary cultures [12], and can provide more material for subsequent culturing and potentially more actively growing and viable tumor cells present in PDX. Thus, PDX generation should be given priority before in vitro expansion of patient material. Major advantages of XLs rely on broad expansion of patient’s tissue in vivo coupled with preservation of intra-tumoral heterogeneity, ease of handling in in vitro conditions, and adequate recapitulation of molecular hallmarks of parental tumors. Because XLs stability in culture is more easily maintained compared to primary cell lines, these models can be used also for genetic manipulation studies. The first comparative study between pairs of CRC primary cultures and XLs was performed in 2007 [112]. Given the reliability of XLs in terms of their retained genomic features compared to PDXs, and their value as predictive drug response models paralleled with in vivo results in different cancer models [114–116], the applicability of this valuable approach has been also tested in CRC with quite a huge success [12, 117]. XLs proved very efficient in describing metastatic inter-lesion heterogeneous patterns correlating with patient lesion-specific response and defined a mechanism of resistance to anti-HER2 therapy in CRC [37].

The value of PDX-derived cell lines has been further underlined by the successful establishment of a cell line bank comprising of 29 CRC XLs with their matched PDX proving conserved distinct genetic, molecular and pharmacologic characteristics [62]. Uncommon molecular subtypes carrying rare genetic lesions may be highly under-represented in common commercial cell line banks, thus constant derivation of new models which may identify novel outliers in response to specific targeted agents is needed. For example, the identification of CRC preclinical models harboring HER2 overexpression due to gene amplification, and the results of anti-HER2 targeted treatment validated in both PDXs and XLs as a valuable tool for genotype-drug correlation studies that have already led to successful clinical trials [55, 56, 118]. At the same time, the proof that specific mutated alleles can represent the mechanism for resistance to anti-HER2 therapy could be provided with XLs that represent more easily manipulable and “editable” models respect to corresponding PDXs [62].

However, there are important limitations of this preclinical model as well that need to be kept in mind. Initially, during the establishment of PDX from patient tumor sample, it is possible that PDX arise as an outgrowth of one or several dominant cell clones, as discussed above. This kind of preselection may lead to inadequate representation of tumor heterogeneity. Looking from the practical point of view as well, in vivo PDXs establishment is money- and time-consuming, and may require in the case of CRC several months.

When looking overall at using 2D cell line models, the artificial aspect lies in passaging for too many generations under in vitro conditions, which over time might lead to the cell line adapting in such a way that it no longer represents the original tumor. Cells also lack the cellular and architectural complexity of patients’ and mice tumors, thus being deficient for the important components of TME. On the other hand, relevant aspects of using XLs in preclinical research include the possibility to derive and expand genotypes which are not commercially available as well as to combine somatic and germline analyses. As XLs are more easily obtained and maintained compared to primary tumor cells, it opens door for performing large-scale in vitro screenings with validation of results in paired PDXs [62].

### Three-dimensional (3D) organoid models

Two-dimensional models do not completely reflect cellular heterogeneity and tissues homeostasis in vivo. Since the discovery of organoids’ culture in 2009 [119] and its application for CRC [120] and for many other tumor types, organoids caused a real revolution in the cancer research field. The technology of growing patients’ tumors as so-called patient-derived organoids (PDOs) under in vitro conditions which mimic their physiological niche allows for the study of biological features of the tumor, the discovery of novel biomarkers, and the monitoring of the response to the treatment in much more cost- and time-effective way compared to PDXs. Additionally, using a sufficient amount of viable material gathered from the tissue biopsy yields establishment success rate around 63% [121, 122].

Since the initial development of the model, different culture methods improving organoid establishment efficiency have been defined [123–125]. Traditionally, intestinal organoids are based on crypt stem cell isolation, but recently a robust strategy of derivation from differentiated human embryonic stem cells or induced pluripotent stem cells was described [126]. Different three-dimensional models, such as spheroids and multicellular aggregates derived from conventional cancer cell lines, or cancer tissue-originated spheroids from primary tumors retaining cell-cell contacts [127] have been applied as patient 3D surrogates in the past. On the contrary, organoids provide three-dimensional cultures comprising multiple cell lineages without immortalization, while keeping the functional, phenotypic and molecular characteristics of the primary tumor [123, 128–130], and the presence of sub-clonal populations preserving the heterogeneity of the tissue of origin [130, 131]. Importantly, cancer gene mutations were described to be stably maintained over relatively long period of continuous in vitro 3D culture [132]. In addition, organoids can be expanded long-term and cryopreserved indefinitely creating living tumor biobanks of colorectal carcinoma of diverse grades and subtypes, adjacent normal mucosa as well as benign tumors [123, 128], heavily-pretreated metastatic CRC [129], sporadic eoCRC [132], liver [133] and peritoneal metastases [134], or precious treatment-naïve locally advanced rectal cancer [122]. Thus, this approach has allowed for in vitro derivation of models which have been strongly underrepresented in cancer research until now. MSI, *BRAF*-mutated, poorly differentiated, and/or of a mucinous type of CRC were on the other hand identified as a hard-to-establish source for PDO derivation [135]. Recently, the development of closer patient-like models went one step further by generating the perfusable mini-gut tubes creating an in vitro tissue (organoids-on-a-chip) with a lifespan of several weeks and ability to regenerate which can also be colonized by microorganisms [136].

As shown throughout the last decade, organoids retain the translational potential to fill the gap between in vitro and in vivo experiments, as well as the laboratory and clinical trials. These models have been used to define cancer vulnerabilities and to improve treatment responses based on their molecular profiling and matching to drug screening results. Thus, organoids coupled with state-of-the-art technology can serve to identify genotype-drug response correlations and prediction biomarkers leading to more rationale-based clinical decisions in the future [131]. Different studies in the latest years showed that CRC PDOs can recapitulate patients’ responses in the clinic and might have predictive value [121, 129, 137]. One paradigmatic example of the utility of PDOs is provided by the cohort of biobanked organoids generated by the Hua laboratory. These models were isolated from treatment-naïve locally advanced rectal cancer patients and were proven to be reliable predictive models for chemoradiation treatment with overall match ratio of 85% [122]. As noted, large cohorts and designed clinical trials will be needed to validate such findings. As such, PDOs open the way to the development of patient tumor-derived programs with the highest co-clinical trials’ potential as predictive models for standard-of-care along with exploration of new off-label drug treatments [138]. Ideally, timeline preclinical testing on PDOs might prevent cancer patients from undergoing ineffective cancer treatment.

Organoids have demonstrated to be suitable models for functional multi-omics analyses [139, 140]. Systematic analyses at genetic, epigenetic, transcriptomic and functional levels of multiple single cell-derived clones of closely related CRC cells of the same tumor displayed extensive intra-tumor diversification with identified mutations being unique to every clone. Furthermore, one interesting study detected marked differences in drugs’ responses among these clones, thus proving organoids reflect intrinsic resistance to treatment [131]. These findings were confirmed by another study showing consequences of intratumor heterogeneity on the growth of tumors and drug response [141]. Separated regions of the primary tumor (called “siblings”) showed distinct functional properties and remarkable genetic heterogeneity with specific mutations. The PDO “sibling” subpopulation determined in vitro to be unaffected by diverse inhibitors was found to form the dominant population when transplanted in vivo in a mixed experiment of treatment-sensitive and -resistant PDOs. Thus, resistant subpopulation escaped drug treatment in vivo, giving rise to a resistant tumor post-therapy and confirming the in vitro observation of PDOs resistance. Mutational patterns and pathways activation in analyzed “sibling” cultures might thus better inform about therapeutic decision-making [141]. In contrast to these studies, Bruun and colleagues [133] compared the pharmacological profiles of PDOs from multiple metastatic lesions and did not observe convincing intra-patient inter-metastatic heterogeneity. This study also suggests that drug response prediction can be improved by expression-based predictive signatures incorporation in addition to genomic markers. In addition, high reproducibility of drug screens using PDOs underscored the unprecedented opportunity of using off-label drugs for patients affected by metastatic CRC, thus opening the possibility for prospective validation in co-clinical trials in the future.

Current approaches in personalized medicine are not only based on testing sensitivities to drugs which are standardly used for the corresponding type of tumor, but also on unravelling specific culprits as novel cancer vulnerabilities, such as definite molecular targets or structural variations. For example, RSPO fusion protein detected in CRC cell lines [142, 143] was also detected in eoCRC PDOs for the first time [132] which might have impact on clinical practice by using Wnt secretion inhibitors or anti-RSPO mAb. Recently, CRC PDOs proved to be useful for studies of dynamic cellular phenotypes within the culture by live-cell imaging and single-cell karyotype sequencing. The data show that multiple genomic instability phenotypes, such as hypermutated MSI and chromosomal instability (CIN), can co-exist, thus potentially opening a therapeutic window for new strategies. For instance, the presence of CIN in late-stage CRC PDOs may render them susceptible to drugs elevating the frequency of chromosome segregation errors [144].

A recent screening with olaparib, a poly(ADP-ribose) polymerase inhibitor (PARP), revealed that CRC PDOs and patient-derived xeno-organoids (PDXOs) represent a functional platform to distinguish between responders and non-responders, and to identify pharmacological correlates for cross-response to different drugs, in this case olaparib and oxaliplatin [94]. This observation suggests that, as it happens in other tissues such as ovarian [145] and pancreatic [146], clinical selection of CRC patients likely to respond to PARP inhibition might be based on sensitivity to previous platinum treatment. In this context, functional screening for PARP inhibitors using PDOs might avoid quality-of-life-harming side effects deriving from prolonged oxaliplatin treatment.

Based on the relevance of EGFR signaling in CRC, new combinatorial strategies with anti-EGFR antibodies are of interest. PDOs served as clinically relevant models to prove the success of concomitant inhibition of KRAS G12C mutation and EGFR blockade which may overcome the resistance of patients to novel KRAS G12C inhibitors in CRC [147]. On the other hand, to test the hypothesis of targeting transient vulnerable state of anti-EGFR treatment-surviving CRC clones with pro-oxidant molecule proved efficient. Combination of anti-EGFR antibody with Vitamin C, a soluble anti-oxidant molecule that can exert pro-oxidant effect when administered at high doses, conducted synthetically lethal metabolic cell death, which might possibly restrict the emergence of acquired resistance to targeted therapy. This approach holds potential to be rapidly translated as innovative treatment in the clinics considering expectation of no added toxicity by Vitamin C [148].

Based on aforementioned findings and many more prior studies, the use of PDOs in cancer research appears auspicious. There are major attributes making organoids highly suitable for drug screening with thoughtful overview of screening protocols being used in different laboratories [149]. As shown, PDOs can be established in a short period, even from needle biopsies, and can be expanded over the long-term into high quantities providing an in vitro platform of clinically relevant models for high-throughput screening. In addition, highly reproducible biobanks can be created in comparison to primary models, with the advantage of providing faster results respect to in vivo screens, capturing patient diversity and predicting their response in the clinic, thus the transition from in vitro to in vivo. Importantly, they can serve as a source for matched in vivo models [123, 129, 137]. PDXs are a great source for in vivo validation of observed in vitro findings as they were proven to yield similar drug responses as their in vitro PDOs counterparts [137]. Moreover, when the patient biopsy specimen is limited, PDXOs can be generated after initial in vivo expansion of patient’s specimen, and still mirror response to clinical treatment [39].

One of the biggest advantages of using PDOs lies in the possibility to preserve tumor microenvironment components during in vitro culture [150]. Since then, more ex vivo protocols were established for co-cultures of mainly lymphocytes efficiently expanded into tumor-reactive T cells together with tumor PDOs to facilitate personalized immunotherapy testing [151]. This platform proved feasible in a co-culture study of autologous peripheral blood lymphocytes as a source of tumor-reactive T cells as an alternative to tumor-infiltrating lymphocytes, and PDOs derived from mismatch-repair-deficient CRC or non-small cell lung cancer patients. This proof-of-principle paper showed a potential to study T-cell mediated PDOs destruction in individualized ex vivo models which should be further explored also in the context of CRC disease [152].

Organoid cultures provide robust assays due to their rapid expansion capabilities. An interesting and promptly growing application is genetic editing of organoids with different applications [153]. PDOs can be combined with CRISPR/Cas9 technology as a powerful approach for modeling of different aspects of cancer research. Genome modifications were used for introduction of driver pathway mutations into human intestinal organoids leading to CRC development [154], mouse model of spontaneous CRC metastasis [155], lineage-tracing experiments of CRC stem cells [156], or to study mutational consequences of DNA repair genes’ knockout in normal colonic organoids [157]. Also, exciting CRISPR screen in human colon organoids [158] or even genome-wide CRISPR screening technology in human intestinal organoids [159] helps to decipher the biology behind CRC, possibly opening way in the future for patient-specific functional genomics. Looking to a bigger picture, panels of organoids with defined mutational profiles might be developed to lead to subsequent identification of a target patient population. Genome editing has been used to develop alterations leading to development of carcinoma with features of serrated CRC [160], as well as preparation of chromosome-engineered human colonic organoids to study the extent of R-spondin gene fusion’s role in tumorigenesis of traditional serrated adenoma [161]. Such rapidly prepared preclinical models allow pharmacological evaluation in this previously untested genetic landscape of cancer with the worst outcomes in CRC patients. Other clinically important example of genes’ cloning into CRC PDOs is the importance of BRAF fusions with different partners in regard to conferred resistance to targeted inhibition of the MAPK pathway, thus suggesting to include BRAF fusions in CRC patients’ genetic screening [162].

In conclusion, organoids at this moment represent one of the closest models to patient’s tumor and, in addition, they can be easily converted into PDX models and vice versa as biological equivalents for matched patient-derived cancer model pairs. Overall, these advantages out-weigh remaining challenges of organoids. As PDOs are an excellent preclinical model for high-throughput drug screens and precision medicine, there is a need to standardize their characterization and procedures to create robust but mainly reproducible methods for their use. One important contributor to quite expensive maintenance of organoids is the need for specific growth factors and inhibitors playing crucial part in mimicking of their natural environment. Once these cost issues will be overcome, organoid technology could be more broadly adopted and likely more organoid biobanks will be available globally. Another highly sought advantage with direct clinical translation is the fact that PDOs can be established from cryopreserved patients’ tissues [163, 164]. Finally, more efforts are needed to improve protocols and conditions to work with tumor-derived organoids in more complex microenvironments including the stromal and immune compartments.

### Preclinical models and their clinical translation

Patient-derived tissue obtained either from surgery or tumor biopsy represents a unique and potentially unlimited source of material for preclinical analyses. Preclinical platforms generated from these samples have reached especially in the last decade a point of development whose relevance is already evident in clinical scenarios, especially in those at early stage, where they have already led to the design of innovative clinical practice-changing CRC trials, as extensively reviewed by [165]. As such, it is of the utmost importance to speed up the translation of rapidly increasing laboratory knowledge into benefits for patients, but at the same time to pay attention to careful data interpretation. Artificial intelligence has been helping to dramatically affect oncology research due to collection of large databases, analyses of which help to uncover relationships among complex biological processes in tumor material or its derived models [166]. The concept of “patient avatar”, intended as the preclinical model derived from a patient and tested with the same treatment as offered to the patient, stands on a growing number of studies where especially PDXs and PDOs have been used to predict the response of individual patients, thus improving treatment outcome and hopefully avoiding unnecessary toxic therapies.

The HERACLES study represents an exemplary proof of the importance of the use of CRC PDXs that served as a discovery and confirmatory preclinical platform for the detection of *HER2* as a novel biomarker for anti-EGFR treatment resistance [55, 57] and the successful transition of these findings into clinical trials which translated into robust improvement in patient outcomes [58, 59, 61, 167]. PDX models have been instrumental also for the study of other mechanisms of resistance to cetuximab treatment. *BRAF* gene alterations are present in around 9% of CRC patients [168], and discoveries on a bench platform put a rationale and stimulated clinical trials’ design where metastatic CRC patients with BRAF V600E-mutated tumors underwent an overall survival benefit and higher response rate from concomitant treatment with BRAFi and MEKi along with anti-EGFR antibody [169]. First exploratory effort few years before using avatar mice from pretreatment core biopsy samples of patients bearing BRAF V600-mutant metastatic disease mirrored the response of patients to selective BRAF and MEK inhibitors [170]. Another highly studied vulnerability in cancer is a specific molecular alteration, the neurotrophic tyrosine receptor kinase *NTRK* gene fusions. Their products, chimeric oncoproteins, are characterized by ligand-independent constitutive activation of the TRK kinase conferring oncogenic potential [171, 172] which was detected also in the CRC [173, 174]. The translation went to development of TRK inhibitors larotrectinib and entrectinib for *NTRK* fusion-positive patients with high response rate success [175]. The following laboratory work was dedicated to identification of resistance mechanisms by analyzing the ongoing treatment with entrectinib in a patient harboring *LMNA-NTRK1* rearrangement by taking advantage of circulating tumor DNA (ctDNA) analyses along with PDX avatar model. The study showed that this powerful preclinical model accompanied by ctDNA samples can identify drug resistance mechanisms in parallel with the clinical treatment of the patients, thus in due time predict resistance and recurrence of the disease [176]. Mice models also proved the mirroring therapeutic response of patients’ clinical outcomes in a retrospective manner where the strong correlation between PDXs with the clinical responses to chemotherapeutic regimens against advanced CRC disease were recapped [177].

Although very perceptive, the couple patient-PDX in co-clinical trial is tremendously resourceful-dependent with the big struggle to reach sufficient scalability to provide pharmacogenomic screens in a high-throughput manner. Zebrafish patient avatars possess strong advantages over mice PDXs in terms of scalability, costs, maintenance and speed of model development with potential to high-throughput automation. So far, many anticancer compounds have been successfully tested in zPDXs, for example, PARP inhibitor combined with DNA-damaging agent temozolomide proved efficacious in combination of elimination of human rhabdomyosarcoma tumor cells. What is more, the consistency of results was shown when the same treatment was applied in mouse xenografts [24]. This study provided the foundation for the initiation of the clinical trial for pediatric rhabdomyosarcoma (NCT01858168), posing a milestone in the use of zebrafish models for preclinical studies. With the matured transplantation protocols, it was possible to start a trial (NCT03668418) which sets a goal to evaluate the predictive power of zPDXs in plethora of malignancies, including CRC.

In this context, also PDOs come to practical usage provided that the establishment, expansion and drug screening can be done in a clinically meaningful time window, thus speeding up the translation from bench to bedside. Even though we still have to improve the successful PDOs establishment rate, this technology allows for the expansion of tumor samples with patient-specific features and potentially broadens the use of FDA- and EMA-approved targeted therapies beyond initially approved indications. Furthermore, one of the major advantages of exploiting PDOs for drug development is the relatively easy establishment also from healthy matched tissue. Such comparisons in testing between tumor- and healthy tissue-derived PDOs hold potential to reduce off-target toxicities from experimental compounds. The first ever reported in-depth study of the similarity between PDOs’ and patients’ actual drug responses dates back to 2018 with the extensive work of the team of Dr. Valeri [129] where PDOs held 88% positive and 100% negative predictive values, suggesting that these preclinical models recapitulated patients’ responses from clinical trials, and could be eventually used for personalized medicine approaches. This pioneering study paved the way for new innovative research endeavors. Already one year later, studies brought the confirming strength of this platform. An observational clinical study named TUMOROID focused on standard-of-care chemotherapeutics in CRC patients succeeded in predicting the outcome of patients to either irinotecan monotherapy or combinatorial use in FOLFIRI setting but failed to foresee the treatment with 5-fluorouracil plus oxaliplatin. Thus, the study points at our limited knowledge of mechanism of action of standard chemotherapy regimen, especially in combinations [121]. Another significant analogy of PDOs’ ex vivo testing with clinical data comes from rectal cancer specimens’ study [178]. PDOs coupled with in vivo testing of radiation and chemotherapy correlated with treatment response in individual patients, more importantly, the testing was performed within a period suitable for potential clinical treatment decision changes. The very similar concept proved also the power of locally advanced rectal cancer PDOs to predict neoadjuvant chemoradiation responses from the clinic with 84.4% accuracy, 78% sensitivity and 91.97% specificity. To highlight again, PDOs results were fully conducted in less than 4 weeks, thus once again strengthened their potential to serve as a “guidance” for therapeutics selection and avoiding of overtreatment [122].

**Fig. 3:**
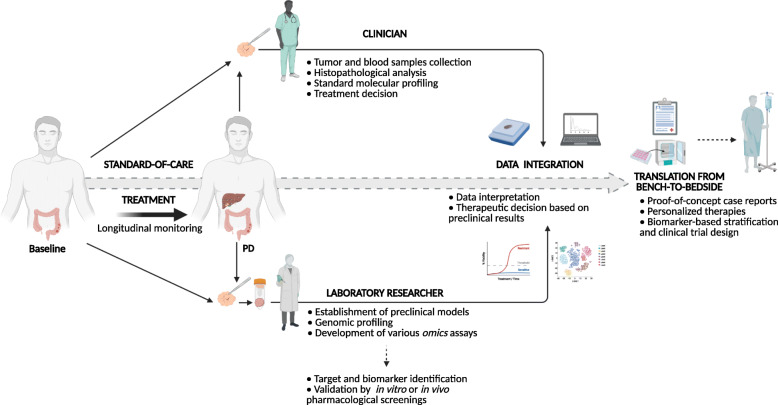
Interconnection between clinics and laboratory leading to precision medicine. The CRC patient is longitudinally monitored from the detection of the disease throughout the standard-of-care treatment. During this time, treatment-naïve tumor samples as well as those from progressive disease can be collected not only for clinical assessment, but also laboratory processing. Analyses from both areas, medical and research oncology, will lead to data integration and interpretation which will finally converge to preclinical evidence-based therapeutic decision-making process. This figure was created with BioRender.com

Still, we are at the very beginning of this exciting era where it seems that PDOs might hold a promising potential to be used as a companion diagnostic tool, but prospective validations in larger cohorts for patient therapy guidance are needed. Also, as a kind of resemblance to liquid biopsy studies, we could think of derivation of PDOs from biopsies collected at time of diagnosis (treatment-naïve, baseline time point) and, in case the patient develops resistance to therapy, at the time of relapse. This might help to better understand the mechanisms behind tumor progression and resistance which could further affect the therapeutic decision-making steps of the clinicians (Fig. 3).

## Conclusion

The ideal preclinical model should accurately mimic the genomic, phenotypic and pharmacologic diversity along with preserved microenvironment of the patient’s tumor, which would make it fitting for testing of novel targets. The extensive research in oncology field to provide suitable preclinical models has been ongoing with the overall vision to revolutionize future healthcare and with the goal of fulfilling personalized medicine for patients. Surgery or bioptic specimens obtained by CRC patients are vital to develop preclinical models and generate complex genetic, transcriptomic and phenotypic characterization suitable for subsequent pharmacological screening and translational studies. Models should be clinically predictive and easily scalable to be flexibly adapted for different applications. Considering all aspects (Fig. 2), organoids will with no doubt continue to be a source of major discoveries in cancer biology and biomarkers field especially because they can serve for middle- to high-throughput screening of different compounds and next-generation cancer treatment. Organoids proves to be an innovative platform for near future of drug discoveries based on their good predictive value and being well-suited for fast, robust, time- and cost-effective using with rapid data generation. Comparing them to other models, organoids better represent tumor heterogeneity and testing can be performed in larger numbers of drug combinations respect to PDX models, thus making them more practical with ethical and effort-related limitations of animal use. In addition, due to their lower cost, higher scalability and potential drug-response predictive ability, zPDXs might represent a novel complementary approach that could speed up the therapeutic decision-making process.

In conclusion, organoids undoubtedly along with patient-derived 2D monolayer cultures, PDXs and zPDXs provide a possibility to integrate multiple *omics* data for precision medicine and open up the opportunity for a preclinical evidence-based patients’ stratification and a more rationale design of innovative clinical trials.

## Data Availability

Not applicable.

## Abbreviations

CCLE: Cancer Cell Line Encyclopedia
CTRP: Cancer Therapeutic Response Portal
CIN: Chromosomal instability
ctDNA: Circulating tumor DNA
CRISPR: Clustered regularly interspaced short palindromic repeats
CRC: Colorectal cancer
CMS: Consensus molecular subtypes
CNA: Copy number alterations
CRIS: CRC intrinsic subtypes
eoCRC: Early-onset CRC
EGFR: Epidermal growth factor receptor
EBV: Epstein-Barr virus
HER2: Human epidermal growth factor receptor type 2
MSI: Microsatellite instability
MSS: Microsatellite stable
Cas9: Native Cas9 nuclease
PDO: Patient-derived organoid
PDXO: Patient-derived xeno-organoid
PDX: Patient-derived xenograft
PARPi: Poly(ADP-ribose) polymerase inhibitor
SNV: Single-nucleotide variant
CAR-T cells: T cells engineered with a chimeric antigen receptor
TME: Tumor microenvironment
XLs: Xenopatient-derived cell lines
zPDX: zebrafish patient-derived xenograft
